# Association Between Nurse Staffing Coverage and Patient Outcomes in a Context of Prepandemic Structural Understaffing: A Patient-Unit-Level Analysis

**DOI:** 10.1155/jonm/8003569

**Published:** 2025-02-24

**Authors:** Maria-Eulàlia Juvé-Udina, Jordi Adamuz, Maribel González-Samartino, Marta Tapia-Pérez, Emilio Jiménez-Martínez, Carme Berbis-Morello, Oliver Polushkina-Merchanskaya, Adelaida Zabalegui, María-Magdalena López-Jiménez

**Affiliations:** ^1^Nursing Research Group, Translational Medicine Area, Bellvitge Biomedical Research Institute (IDIBELL), L'Hospitalet de Llobregat, Catalonia, Spain; ^2^Department of Nursing Management, Catalan Institute of Health, Barcelona, Catalonia, Spain; ^3^Department of Fundamental and Clinical Nursing, Faculty of Nursing, University of Barcelona, L'Hospitalet de Llobregat, Catalonia, Spain; ^4^Nursing Knowledge Management and Information Systems Department, Bellvitge University Hospital, L'Hospitalet de Llobregat, Catalonia, Spain; ^5^Infectious Disease Department, Bellvitge University Hospital, L'Hospitalet de Llobregat, Catalonia, Spain; ^6^Department of Nursing, Joan XXIII University Hospital, Tarragona, Catalonia, Spain; ^7^Department of Nursing, Faculty of Nursing, Rovira i Virgili University, Tarragona, Catalonia, Spain; ^8^Department of Nursing, Hospital Clínic, Barcelona, Catalonia, Spain; ^9^Nursing Research Group, Interdisciplinary Research Area, August Pi i Sunyer Biomedical Research Institute (IDIBAPS), Barcelona, Catalonia, Spain

## Abstract

**Objective:** To evaluate the association between nurse staffing coverage and patient outcomes in a context of structural understaffing.

**Design:** This is a population-based, cross-sectional, multicenter study, including patient and staffing data from eight public hospitals from Catalonia, Spain.

**Participants:** A total of 183,085 adult in-patients admitted to hospital wards and step-down units during 2016 and 2017.

**Outcomes:** In-hospital mortality, 30-day hospital readmission, and three cluster nurse-sensitive adverse events: healthcare-acquired infections, failure to maintain, and avoidable critical complications. The study factor is safe nursing staffing equivalent to nurse staffing coverage > 90%.

**Results:** Average patient acuity was equivalent to 4.5 required nursing hours per patient day. The mean available nursing hours per patient day was 2.6. The average nurse staffing coverage reached 65.5%. Overall, 1.9% of patients died during hospitalization, 5% were readmitted within 30 days, and 15.9% experienced one or more adverse events. Statistically significant differences were identified for all patient outcomes when comparing patients safely covered (nurse staffing coverage > 90%) and under-covered (nurse staffing coverage < 90%). Increasing nurse staffing coverage to a safe level (> 90%) is associated with a reduction of the risk of death (RR: 0.41, 95% CI: 0.37–0.45), a decrease in the risk of hospital readmission (RR: 0.93, 95% CI: 0.89–0.97), and a reduction of nurse-sensitive adverse events (RR: 0.67, 95% CI: 0.66–0.69).

**Conclusion:** Safe nurse staffing coverage acts as a protective factor for detrimental patient outcomes, significantly reducing the risk of in-hospital mortality, 30-day hospital readmission, healthcare-associated infections, failure to maintain, and avoidable critical complications. Further policy efforts are needed to guarantee a safe registered nurse staffing coverage.

## 1. Introduction

Reducing avoidable mortality, hospital readmissions, and adverse events is a top priority to promote patient health and recovery, improve quality of care, enhance patient safety, and contain healthcare costs. Evidence on the association between nurse–patient ratios and patient mortality, length of stay, and other outcomes has been published in the last decades [1–6]. Missed nursing care has been found to act as a mediator of this relationship [7, 8], and the work environment has also been identified to influence patient outcomes [9–11]. Moreover, the prevalence of older people with multimorbidities and disabilities has increased in the last decades [12], while nurse shortages persist in the healthcare systems [13]. Increasing service demands further amplify the existing chronic nurse understaffing [14] while staffing ratios widely differ within and among countries, hospitals, units, and shifts [4, 15, 16].

Multiple inquires show the impact of nurse staffing levels (patient-to-nurse ratio) on the quality of patient care [17]. Despite this evidence, lack of consensus exists on what is and how to determine safe staffing levels in nursing [14, 18]. While several patient classification systems and methodologies to cluster patients based on nursing care requirements have been developed, the need to consider individual patient acuity in the staffing equation and to determine the outcomes relate to staffing still remains [19–21]. In this sense, the dose and administration of nursing care, related to total nursing hours per patient day, to determine the difference between the available and required number of registered nurse (RN) hours to sufficiently cover patient care demands, have been scarcely mentioned in the nursing literature in an explicit manner [14, 22]. Pragmatically, structural nurse understaffing could be understood as sustained nurse staffing below the minimum recommended, but the minimum recommended depends on patient acuity in terms of the number of RN hours required. Recent studies consider understaffing when below the average of the usual staffing in the wards [23] or at a cutting point below 80% of the median nursing staffing in the units [24]. Nevertheless, this conceptualization might not apply in practice settings experiencing chronic structural nurse understaffing. In addition, the results from Griffiths et al. [23] demonstrating a U-shaped relationship between nurse assistants (NAs) and inpatient mortality, and a lack of top of the curve effect for RN, pose further questioning on how to define standards for minimally appropriate staffing levels [19]. Interestingly, Park's Optimized Nurse Staffing Estimation Theory contributes to determine the maximum quality of care for patients while simultaneously delivering nurse staffing in the most cost-effective way, therefore illustrating that optimal and safe staffing are not equivalent [25]. In the words of the author “this innovative multi-dimensional econometric theory also allows to determine the Optimized Nurse Staffing (Sweet Spot) and the Optimum Nurse Staffing Zone to mediate between nurse and stakeholders and establish an evidence-based economical goal to hire a sufficient number of nurses to guarantee reasonable patient care.” Park's theory (2017), unlike other nursing workforce studies, is noteworthy for being flexible in terms of input variables such as the number of nurses, nursing care hours, and/or skill mix; even so, this theory still needs further validation using empirical data.

In this respect, different patient classification systems have been developed to measure nursing workload; however, only the acute to intensive care (ATIC) patient classification system measures patient acuity based on the weight of the patient main problem identified in the nursing care plan, equivalent to required nursing hours per patient day (*r*NHPPD) [26]. Nurse staffing coverage embeds the balance between RN hours required by each patient to meet their safety needs and minimize deleterious health outcomes and the available or real offered RN hours to each patient [27]. The difference between available and actually provided RN hours lies in the fact that the former is a staffing unit-based metric, whereas the latter is an individual-based variable measurable only at bedside, after nurse–patient assignment, and formal or informal reassignments according to their progress. Previous studies conclude that two-thirds of adult patients admitted in hospital wards call for more intensive than acute care, requiring an average of 5.6 RNHPPD, and this is associated with poor patient outcomes [26, 28]. The mean nurse–patient ratio of 1:6 for patients in hospital floors and the need to move downwards 1:4 are frequently mentioned in the staffing literature from North America or Australia, although in many other regions of the world, these ratios are largely away from the actual mean ones [4, 29].

On the other hand, nurse-sensitive outcomes were defined as relevant result measures that reflect changes in the health of patients that are directly affected by nursing care [30] like mortality or hospital readmission. Despite the efforts of several professional organizations and agencies, no consensus on which outcomes are considered sensitive to nursing practice and no standardized definitions of their measurement have been achieved yet.

This study was aimed at evaluating the association between nurse staffing coverage and patient outcomes in terms of in-hospital mortality, 30-day hospital readmissions, and cluster nurse-sensitive adverse events, including healthcare-acquired infections, failure to maintain, and avoidable critical complications, in a context of a structural RN understaffing before the onset of the COVID-19 pandemics.

## 2. Methods

### 2.1. Study Design and Setting

This is a population-based, cross-sectional, multicenter design, including patient and staffing data from 2016 to 2017. This study was conducted in a public hospital system in Catalonia (Spain) including three hi-tech metropolitan facilities, three urban university centers, and two community hospitals, accounting for more than 3000 beds, 145 wards and step-down units, and 130,000 annual patient discharges. The average nurse per patient ratio in adult hospital floors was 1:10.5 (6–18) and 1:4 (3–6) in step-down units. Study approval was granted by the institutional research ethics committee (PR385/18).

### 2.2. Participants

Eligible participants were all those aged 18 or older, admitted to wards and step-down units. Obstetrics, maternal–child, and pediatric patients were excluded. Observation units were not included either. The study was intended to consecutively include all admitted patients matching selection criteria. This represented an initial sample estimation of 200,000 adults.

### 2.3. Data Sources

Patient data were obtained from the electronic health record system, the hospital minimum dataset, and the clinical data warehouse. Using the patients' unique identification number, datasets from these sources were linked. Nurse staffing data were obtained from nursing human resource databases and ward structural assignment reports.

Merged patient data were subsequently matched to staffing data, considering the unit and the time frame each patient received care within.

### 2.4. Measures

#### 2.4.1. Patient Acuity and Required RN Hours per Patient day

Patient acuity measurement was based on the ATIC patient classification system that clusters acuity into 10 categories of nursing intensity, equivalent to required RN hours per patient day (*r*NHPPD) and to nurse per patient ratio (Appendix 1). This system showed sound capacity to discriminate patient acuity [26].

#### 2.4.2. Available RN Hours and RN Staffing Coverage at Unit Level

Nurses were grouped into a single category, with a minimum of university bachelor's degree mandatory for RN practice in the context of the study. Nursing supporting staff, licensed practical nurses (LPNs), or NA were not considered.

Available RN hours were divided by the total number of patients in the units every day to generate the daily available RN hours per patient day (*a*NHPPD), and then they were aggregated to unit-shift and unit-day level.

The difference between *a*NHPPD and *r*NHPPD was used as a measure of the balance of daily NHPPD. Nurse staffing coverage was defined as the proportion of RN *r*NHPPD reached by the *a*NHPPD.

#### 2.4.3. Patient Outcomes

• In-hospital mortality included patients deceased during hospitalization as coded on patient hospital discharge report [3].
• 30-day hospital readmission accounted for the number of patients who experienced unplanned, all-cause, acute hospital admission, within 1–30 days after discharge from hospitalization.
• Nurse-sensitive adverse events. This study operationalized nurse-sensitive adverse events as the occurrence of one or more of the outcomes categorized in three clusters: healthcare-associated infections, failure to maintain, and avoidable critical complications, not present on admission day (Appendix 2 details terminology codes).
  i. Healthcare-associated infections. Surveillance definition considers infections due to cross-contamination between patients and healthcare workers, physical environment, facility waste management, or due to medical devices, occurring after the second day of hospitalization [31]. This study included surgical site infection (SSI), peripheral and central-line-associated bloodstream infection (CLABSI), catheter-associated urinary tract infection (CAUTI), and hospital-acquired infections requiring isolation precautions for multidrug-resistant organisms (MROs).
  ii. Failure to maintain. Defined as cascade-effect complications deriving from patient functional and cognitive decline, the proposal for this quality indicator included pressure injuries, pneumonia, urinary-tract infection (UTI), and delirium [32]. This inquiry accounted hospital-acquired failure to maintain complications, not present on admission, that may persist at discharge, including pressure injuries, delirium, aspiration pneumonia, hypostatic pneumonia, persistent incontinence, venous catheter-related phlebitis, and falls with or without injury.
  iii. Avoidable critical complications. This cluster included those cascade-effect, life-threatening complications dependent on missed nursing care, nurses' early recognition, escalation, and decision-making [33], considering cardiac arrest, shock, thrombotic events, and sepsis, not present on the admission day.

### 2.5. Data Analysis

Baseline characteristics of participants were described using mean and standard deviation for continuous variables and frequencies for categorical variables.

Safe nurse staffing coverage threshold was based on the incidence curves of the main outcomes, employing range estimates of lower incidence points resulting in > 90%.

Risk factors found in previous studies were considered as covariates [3]. Patient features for the adjustment of outcomes included age, gender, admission type (planned or unscheduled), reason for admission, and clinical assignment (medical or surgical). Hospital characteristics considered unit level (ward or step-down unit) and hospital level (hi-tech or other).

A modified Poisson regression with mixed effects and robust error estimates by the sandwich method was used to assess the association between nurse staffing coverage and patient outcomes. Use of mixed models was necessary to account for correlation between participants within hospital. Potential confounders considered for adjustment in the multivariate models included previously mentioned covariates. Risk ratio (RR) and 95% confidence intervals were estimated to assess the association while illustrating the adjusted effect of the study exposure. All statistical analyses were conducted using R version 3.5.1.

## 3. Results

Initial study population accounted for 199,761 adult inpatients. The final analysis excluded 16,084 of cases due to inconsistencies, duplicates, or absence of data to determine patient acuity (8.3%). Patients identified as close to the end of life at point of admission were excluded, given its close relationship with mortality and other study outcomes (*n* = 592).

A final sample of 183,085 participants were considered for analysis, accounting for 1,265,068 patient-days. The patients' mean age was 64.1 years (SD 17.4); most were male adults (56%), admitted to high-tech hospitals (56.8%), in medical wards (52.2%), with unscheduled admission (55.3%). The proportion of patients with high or very high APR-DRG severity and risk of mortality [26] was 23.5% and 17.5%, respectively. The most frequent reasons for admission were cardiocirculatory conditions (16.6%) and infections (14.9%) (Table 1).

### 3.1. Patient Acuity and Staffing Measures

Table 2 details patient acuity, staffing measures, and outcome descriptive findings.

Almost two-thirds of inpatients (64.8%) were clustered into more intensive than acute care acuity groups, mostly focused on the intensification and intermediate categories (3.5–7.0 *r*NHPPD). The average patient acuity was equivalent to 4.5 *r*NHPPD (SD 1.7). The mean *a*NHPPD varied among hospitals and units, from 1.6 in a medical unit to 6.0 *a*NHPPD in most step-down units. The average *a*NHPPD was 2.6 (SD 0.9).

On average, nurse staffing coverage was 65.5%, ranging from lower than 30% to higher than 90%. The mean nurse staffing coverage was higher in high-tech hospitals (71.8%) when compared to other facilities (58.1%).

### 3.2. Patient Outcomes

Overall, 3419 patients died during hospitalization (1.9%), 9214 were readmitted within 30 days (5%), and 29,158 experienced adverse events (15.9%).

Mortality was lower in hi-tech hospitals when compared to the other facilities (1.7% vs. 2.0%), as well as 30-day hospital readmission (4.7% vs. 5.2%). Conversely, adverse events were more frequently documented in hi-tech facilities (16.5% vs. 15.1%) (Table 2).

Patients in the preintensive group had higher 30-day readmission and mortality values (9.8% and 5.5%, respectively) than those in the intermediate category (7.0% and 3.3%, respectively). While the incidence of adverse events was almost identical in both groups (26.9% vs. 26.6%), patients in the preintensive cluster had a higher frequency of failure to maintain (20.9% vs. 16.0%) and lower incidence of healthcare-associated infection (7.5% vs. 10.3%) and avoidable critical complications (3.3% vs. 7.0%).

When clustering patients according to their reason for admission, the average nurse staffing coverage was below the overall mean (65.5%) in patients admitted for digestive conditions (mean nurse staffing coverage 58.1%), infections (43.0%), respiratory diseases (45.0%), hematologic and immunologic disorders (43.8%), and other conditions (35.6%) (Table 3).

### 3.3. Association Between Safe Nurse Staffing Coverage and Patient Outcomes

The incidence curve for outcomes at different levels of nurse staffing coverage exhibits decreasing trends as the nurse staffing coverage increases. Lower incidence values are observed at nurse staffing coverage between 75% and 125% for mortality, 85%–110% for a 30-day hospital readmission, and 90%–100% for adverse events. These values orient safe nurse staffing coverage at a target point of over 90% (Figures in Appendix 3).

Statistically significant differences were identified for all patient outcomes when comparing patients safely covered (nurse staffing coverage > 90%) and under-covered (nurse staffing coverage < 90%), except for the case of cardiac arrest (*p*=0.15) and surgical site infection (*p*=0.40) (Table 4).

Multivariate analysis demonstrates the association between safe nurse staffing coverage and patient outcomes (Table 5). After adjusting the regression model for covariates, the RRs of mortality and 30-day hospital readmission were 0.41 (95% CI: 0.37–0.45) and 0.93 (95% CI: 0.89–0.97), respectively. The RRs of adverse event were 0.67 (95% CI: 0.66–0.69), in terms of healthcare-associated infections (RR: 0.60, 95% CI: 0.57–0.63), failure to maintain (RR: 0.69, 95% CI: 0.67–0.72), and avoidable critical complications (RR: 0.46, 95% CI: 0.43–0.49).

## 4. Discussion

This study examined the association among nurse staffing coverage, the proportion of *r*NHPPD reached by the *a*NHPPD, and in-hospital mortality, 30-day hospital readmission, and three cluster adverse events in a context of structural understaffing, before the onset of the COVID pandemics.

The main finding of this inquiry is that safe nurse staffing coverage (> 90%) is associated with a significant reduction in the risk of inpatient mortality, 30-day hospital readmission, and adverse events. RR values in the adjusted regression model indicate that safe nurse staffing coverage acts as a protective factor for all analyzed outcomes. These results provide additional support to the body of research demonstrating a positive relationship between appropriate nurse staffing and a decreased risk of mortality, hospital readmission, and adverse events. Our findings could be considered an empirical testing approach of the Park's Optimized Nurse Staffing Estimation Theory [25] in the hospital setting.

Life expectancy at age 60 in the country of this study is one of the highest in the world [34]. Standardized death rates from chronic diseases are among the lowest in Europe [35]. Inpatient mortality rates in the region (3.2%) are the lowest at the national level (4.2%) and below the mean of the EU (4.0%) [36–38]. Inpatient mortality incidence in this study (1.9%) is coincident with the values reported by Needleman et al. [3].

Our findings align with other previous inquiries on the association between nursing staffing and 30-day mortality [1, 4], as well as with inpatient mortality, found to be significantly associated with nurse understaffing [3]. While a previous study found no top of the curve for the association between RN staffing and mortality [23], our findings draw a U-shaped curve; however, the differences on the study designs, staffing values (mean *a*NHPPD 4.7 vs. 2.6), and mortality measures must be considered. To what extent different levels of RN staffing are associated with mortality is still unanswered.

In this study, overall, a 30-day hospital readmission incidence was 5%, with higher readmission rates for patients admitted due to advanced oncologic conditions, hematologic and immunologic disorders, digestive, kidney, and infectious diseases, similar to the most frequent 30-day all-cause hospital readmission groups by principal diagnosis at the index admission reported [39]. Nevertheless, readmission rates in our study are below these updated values and the ones referred in other reports [39, 40]. This might be probably related to the fact that this study included any all-cause acute readmission within 30 days of discharge, not within 30 days of discharge from the admission index [41]. In addition, our study was conducted in a public healthcare system within a universal health coverage model, substantially different from that of the report referred to Medicare, Medicaid, uninsured, and private insured patients in the US [39, 42].

Hospital readmission has been related to substandard care, represents a burden for patients, and increases costs. Several inquiries found an association between 30-day hospital readmission with nursing staffing, with high-level nurse staffing hospitals being more prone to avoid this outcome [43–45]. One of them, with a large sample of chronic respiratory patients, found that a higher number of RN was associated with a lower risk of readmission rates (7.9%–8.9%) [45]. With caution, this finding might be considered consistent with our results. Additionally, patient readiness for discharge, an outcome within the scope of professional nurses' accountability, may be a related measure but it was not considered in our study. Poor readiness for discharge was identified as an individual complexity factor [46], and it was related to early readmission [40, 41].

Furthermore, previous studies have related nursing staffing to several adverse events, including failure-to-rescue [9, 47, 48], cardiac arrest [49], medication errors [50], and healthcare-associated infections [27]. In the same direction, worse nursing staffing is associated with higher risk for patients and therefore is an indicator of a hospital's capacity to provide quality nursing care [51].

A group of largely preventable complications, healthcare-associated infections are mostly caused by less than 20 microorganisms, among which 20% exhibit multidrug-resistant phenotypes [52]. Prevalence is estimated at 9%–20%, while incidence is estimated at 5%–10% [52–54]. At the cluster level, this study considered not present on admission SSI, CLABSI, CAUTI, MRO, and aspiration pneumonia, with an overall frequency reaching 5.5%.

Overall, a low incidence of healthcare-associated infections in this study is probably related to the use of hospital minimum dataset as source data for these measures, suggesting poor discharge coding of these events at the administrative data level. Cautionary notes have been raised in the specialized literature regarding the limited and highly variable accuracy of administrative data for the purposes of healthcare-associated infection surveillance [55], an issue that must be acknowledged as a significant limitation in our study. Notwithstanding this gross approach, the results of this inquiry align with previous studies demonstrating that increased staffing is related to a decreased risk of healthcare-associated infections [27, 56].

Similarly, according to the findings, nurse staffing coverage under 90% is significantly associated with failure to maintain complications, which are potentially preventable with basic nursing care interventions and are known to occur as a consequence of care rationing [33]. Worse nursing staffing was associated with basic nursing care left undone and higher mortality rates [8, 57]; however, no previous clinical studies on the association between nurse staffing and failure to maintain have been found, neither for the association between nurse staffing and aspiration or hypostatic pneumonia, delirium, and persistent incontinence. Contradicting results are observed for the association between nurse staffing and pressure ulcers, while higher nurse staffing has been strongly associated with fewer inpatient falls [58, 59]. Only a few previous studies on the association between nurse staffing and infusion phlebitis were located [28, 60].  Appendix 4 summarizes additional considerations on the detailed adverse events.

On the other hand, the association between nurse staffing on failure to rescue was demonstrated in previous studies [9, 47]. Similarly, evidence assessing nursing surveillance on survival after cardiac arrest and the effect of better nurse staffing on this outcome has been published [49, 61]. This inquiry was intended to assess the association between nurse staffing coverage and life-threatening complications, potentially preventable with proper surveillance, early recognition, escalation, and decision-making. Frequency values of shock and cardiac arrest in our study (1.2%) are quite consistent with the incidence rates reported in a previous one (1.4%), while values are significantly higher for sepsis (1.8% vs. 0.9%) and lower for thrombotic events (0.6% vs. 1.3%) [48]. Multivariate analysis findings demonstrated a strong association between safe nurse staffing coverage and a decreased risk of avoidable critical complications, aligning with the results of the mentioned study, evidencing significant association for RN staffing and shock, cardiac arrest, and other outcomes [48, 62].

### 4.1. Strengths and Limitations

The strengths of this study include its large sample size, the identification of patient acuity distribution, and the measure of nurse staffing coverage reflecting the balance between *r*NHPPD and *a*NHPPD. Our findings on the association among nurse staffing and in-patient mortality, hospital readmission, healthcare-associated infections, and avoidable critical complications reinforce the existing evidence and align with Park's Optimized Nurse Staffing Estimation Theory [25]. In addition, this is the first study demonstrating a statistically significant association between nurse staffing and failure to maintain as a cluster nurse-sensitive outcome.

Inquiries on the relationship between nurse staffing and patient outcomes use nurse–patient ratio, full-time equivalents, skill-mix, nurse-perceived staffing adequacy, or nurse-reported number of assigned patients as staffing measures; however, NHPPD has been identified as the most reliable and frequently used variable [63]. This study employed nurse staffing coverage as a derivate measure of NHPPD and a patient classification system that, despite some acknowledged limitations [26], exhibits remarkable predictive ability for patient acuity [26, 28].

Similar to recent studies, this inquiry did not include NA hours, considering these healthcare workers as a complement, not a substitute for RN [23, 48]. The idea that some patient care can be provided by NA without affecting patient outcomes is more an assumption than an evidence-based fact, and probably, chronic RN understaffing have fed this widespread belief. On the contrary, prior studies have demonstrated the effect of richer skill-mix, or higher proportion of RN, and a reduced risk of mortality and other quality indicators [64]. Even for those adverse events that could be assumed as a potentially sensitive to NA, since they relate to basic nursing care, avoiding cascade-effect complications from functional and cognitive decline required not only performance skills but also clinical judgment abilities acknowledged for RN, not for NA.

On the other hand, advancing that 90% target point for safe nurse staffing coverage could be considered arbitrary [19], this value was set based on the incidence curves of the main outcomes. A high proportion of patients (79.2%) fell into the unsafe nurse staffing coverage group, indicating the existence of structural nurse understaffing in the context of the study. Furthermore, the average *a*NHPPD was significantly lower when compared to that reported by Griffiths et al. (2.6 vs. 4.7) [23]. Existent structural nurse understaffing would prevent the use of other measures for determining understaffing, such as staffing below the usual mean or median [23, 27].

In addition to those implicit in a cross-sectional design, several limitations should be considered when interpreting the results of this study.

First, acuity was measured based on the patient main problem identified by RN in the patient first care plan, ruling out subsequent changes. Since patient needs may change over time and are not lineally distributed across days [14, 65], a longitudinal design would increase accuracy. In this same sense, *a*NHPPD in this study is not actually provided RN HPPD but available at the unit level. The role of RN prioritization and actual time devoted to each patient has not been explored. In this sense, previous studies showed that nurse turnover, nurses' extended work hours, and floating staff between units and temporary hires were associated with nurse and patient outcomes [66–68]. To date, most of the nursing patient classification systems are based on scores that measure selected nursing tasks. They may explain the nursing workload, but they are not predictive of acuity according to the patient needs [69]. The ATIC patient classification system used in our study allows us to determine patient acuity, according to main nursing diagnoses in terms of *r*NHPPD. This system revealed notable predictive ability for patient acuity [26]; it is implanted in the managerial daily practice [70] and has been used in a previous study to demonstrate the association of nurse staffing coverage levels and selected patient outcomes [28]. Although we are not knowledgeable about previous studies that used patient classification systems based on nursing diagnoses, our methods concur with previous inquires that used patient workload scores that consider not only patient-related tasks but also psychosocial status and nursing care plans of electronic health records [71, 72].

Second, outcome data were collected from the electronic health record and the hospital's minimum datasets during a prepandemic period. Although these data sources might be considered more objective than surveyed or nurse-reported data, concerns on administrative data use for research purposes have been raised [2, 48, 55]; thus, adverse event rates in this study are probably underestimated. This is an acknowledged limitation in similar studies [50]. To minimize this effect, adverse event data were collected from the source that might provide more accurate data, based on a previous study where RN demonstrated higher accuracy than physicians at diagnosing and charting pressure injuries, and similar accuracy for SSI, while physicians exhibited higher accuracy for the diagnosis and documentation of aspiration pneumonia [73]. As referred in the literature, it must also be acknowledged that adverse event data from high-tech hospitals in this study were more accurate than data from other facilities [48]. Potential confounders related to patient and hospital characteristics were included for adjustment in the multivariate models. Although patient comorbidities were not considered in this inquiry, we have included the reason for admission, unit level, and clinical assignment. Moreover, future studies should evaluate whether nursing coverage and patient health outcomes improve after staffing policy effort implementation.

Third, the study considered not present on the admission day instead of after the second day of hospitalization occurrence outcomes; this is a significant limitation, mostly for healthcare-associated infections.

Fourth, prior evidence legitimates mortality, failure to rescue, 30-day hospital readmission, and healthcare-associated infections as nurse-sensitive outcomes. Notwithstanding, there is a need to advance in the clarification of detailed coding and univocal identification of the content of each adverse event, with particular focus in those specific adverse events in the junction between two or more clusters, such as the case of aspiration pneumonia or UTI, that can be classified into the healthcare-associated infections or the failure to maintain clusters. Similarly, sepsis has been previously grouped as a healthcare-associated infection [27] and an avoidable critical complication [48]. Peripheral-venous catheter phlebitis was not originally included in the failure to maintain construct [33], neither was applicable as a healthcare-associated infection or an avoidable critical complication, even though it is a known risk factor for both line-associated bloodstream infection [74] and line-associated thrombotic events [75, 76]. Clustering adverse events in this study was based on the available literature and, ultimately, on the authors' consensus. Refined detailed adverse events are also needed to advance in cost estimates.

### 4.2. Implications for Nursing Management

Our study shows the association of safe nurse staffing coverage and health outcomes using the ATIC patient classification system, measuring patient acuity based on the weight of the patient main problem, equivalent to *r*NHPPD. From a managerial perspective, the innovative multi-dimensional econometric theory posed by Park may reach a sense far beyond home healthcare, as our methods and findings on inpatients align with the essential concepts of this theory [25]. Evidence on strategies for implementing viable safe nurse staffing policies in practice is still scarce [77]. This study shows the association of nursing coverage and patient outcomes using a target point of safely covered > 90% and provides a tool to determine safety nursing coverage, according to patient needs that might be used to inform policymakers and assist nurse managers' decision-making, while considering the optimum nurse staffing zone proposed by Park [25].

Up to now, nursing staffing ratios have not improved despite mainstream nursing workforce research and the impact on patients' health outcomes [6, 77, 78]. The use of theory-based tools to determine nurse staffing needs according to the complexity and acuity of patients should be encouraged in daily managerial practice to ensure patient safety and quality of care. Managers' education on nurse staffing theory concept understanding and its translation into applicable strategies for practice is probably needed.

The current shortage of nurses in the healthcare systems is a priority in the nursing agenda, although it may not be for some policymakers [13]. Then, nurse executives' strategic investments may lead to improved health workforce planning and probably will be pivotal in implementing health policy reforms and anticipating future population needs. In fact, the strategic plan of the Catalan Institute of Health provides a policy oriented to progressive appropriateness nursing staffing in hospital patient wards based on safety nursing coverage [70].

Finally, many patient complications might be occurring in a cascade sequence originated with missed basic nursing care, either related to structural nurse understaffing or to task-oriented care delivery models. In the latter case, nursing care provision is managed by means of patient care assignment division between RN and NA, instead of in a patient-centered approach, where the RN is accountable for all nursing care and NAs have a complementary role. In the setting of this inquiry, both models coexist [70], with ward teams fully committed to promote nurse autonomy and a patient-centered approach and units still functioning under a Taylorian-like task-approach [79]. The significant contribution of Park's theory is acknowledged, showing the relationship between minimum and optimal nursing staffing [77], challenging further studies contributing to a deeper understanding on the threshold of professional nurse staff and complementary staff needed to achieve optimal outcomes [19], as well as studies on staffing and care left undone and on NA patient care left unsupervised in task-oriented care delivery settings, while shifting to upgrade the work environment, and increase RN staffing to improve patient outcomes.

## 5. Conclusion

In a context of prepandemic structural understaffing, nurse staffing is significantly associated with nurse-sensitive outcomes. Safe RN staffing coverage (> 90%) acts as a protective factor for detrimental patient outcomes, significantly reducing the risk of in-hospital mortality, 30-day hospital readmission, and cluster nurse-sensitive adverse events in terms of hospital-acquired infections, failure to maintain, and avoidable critical complications in adult inpatients. Immediate future policy efforts are needed to guarantee safe nurse staffing coverage.

## Figures and Tables

**Table 1:** Baseline sample characteristics.

Characteristic	Study population (*n* = 183,085)	
	No	(%)
Demographic characteristics		
Age (years)_mean (SD)	64.1	(17.4)
Age ≥ 75 years	57,523	(31.4)
Male sex	102,486	(56)
Medical ward	95,491	(52.2)
Psychiatric ward	608	(0.3)
Surgical ward	87,594	(47.8)
Step-down unit	13,559	(7.4)
Unscheduled admission	101,169	(55.3)
Length of stay_mean (SD)	6.9	(9.3)
Discharge to another facility	7328	(4)
Severity (APR-DRG 3-4)	43,095	(23.5)
Mortality risk (APR-DRG 3-4)	32,084	(17.5)
High-tech hospital	103,958	(56.8)
Reason for admission		
Cardiocirculatory	30,336	(16.6)
Infectious	27,208	(14.9)
General surgery	20,766	(11.3)
Trauma and orthopedics	19,951	(10.9)
Digestive, liver, and pancreatic	1979	(10.8)
Nervous system	15,472	(8.5)
Kidney and urinary tract	13,959	(7.6)
Respiratory	10,971	(5.6)
Reproductive	8257	(4.5)
Head, neck, and maxillofacial	5501	(3.0)
Metabolic, nutritional, and endocrinology	3064	(1.7)
Hematopoiesis, blood, and immunologic	2705	(1.5)
Psychiatric, mental health, and addictions	1192	(0.7)
Skin and burns	907	(0.5)
Eyes	857	(0.5)
Other conditions	2159	(1.2)

*Note:* Other condition groups mainly included admissions for unspecific clinical decline related to advanced neoplastic disease in fragile adults.

Abbreviations: *a*NHPPD = available RN hours per patient day, APR-DRG = all patient refined diagnosis-related groups, IQR = interquartile range, *r*NHPPD = required RN hours per patient day, SD = standard deviation.

**Table 2:** Acuity clusters according to the ATIC patient classification system, nurse staffing coverage, and patient outcomes.

Features and outcomes	All		Acute		Intensification		Intermediate		Preintensive		Intensive		Super-intensive	
	*n* = 183,085		*n* = 64,403 (35.1)		*n* = 54,059 (29.5)		*n* = 50,803 (27.7)		*n* = 13,376 (7.3)		*n* = 363 (0.2)		*n* = 81 (0.04)	
	*N*	(%)	*N*	(%)	*N*	(%)	*N*	(%)	*N*	(%)	*N*	(%)	*N*	(%)
Clinical characteristics														
Age (years)_mean (SD)	64.1	(17.4)	59.7	(17.0)	63.7	(17.4)	67.8	(16.8)	73.3	(13.9)	63.4	(16.8)	62.8	(12.7)
Age ≥ 75 years	57,523	(31.4)	13,400	(20.8)^a^	16,357	(30.3)	20,458	(40.3)	7187	(53.7)	105	(28.9)	16	(19.8)
Male sex	102,486	(56)	32,147	(49.9)	32,885	(60.8)	29,428	(57.9)	7752	(58)	215	(59.2)	59	(72.8)
Severity or mortality risk 3–4	47,572	(26.0)	3256	(5.0)	11,850	(21.9)	22,985	(45.2)	9081	(67.9)	321	(88.4)	79	(97.5)
Surgical ward	87,594	(47.8)	57,085	(88.6)	21,463	(39.7)	821	(16.2)	618	(4.6)	153	(42.1)	65	(80.2)
Step-down unit	13,559	(7.4)	2386	(3.7)	3410	(6.3)	7356	(14.5)	330	(2.5)	29	(8)	48	(59.3)
Prior ICU admission	10,880	(5.9)	1267	(1.9)	3560	(6.6)	4985	(9.8)	781	(5.8)	221	(60.9)	66	(81.5)
Discharge to another facility	7328	(4)	1539	(2.4)	1720	(3.2)	33	(6.5)	726	(5.4)	26	(7.2)	17	(21)
Length of stay_mean (SD)	6.9	(9.3)	3.7	(6.0)	7.3	(9.2)	9.5	(10.7)	9.6	(9.4)	23.6	(26.4)	67.4	(46.1)
High-tech hospital	103,958	(56.8)	37,948	(58.9)	30,200	(55.9)	28,531	(56.2)	6969	(52.1)	245	(67.4)	65	(80.2)
Nurse staffing coverage														
*r*NHPPD_mean (SD)	4.50	(1.7)	2.7	(0.1)	4.1	(0.5)	6.1	(0.5)	7.9	(0.5)	11.6	(0.5)	14	(0.0)
*a*NHPPD_mean (SD)	2.59	(0.9)	2.5	(0.7)	2.6	(0.8)	2.8	(1.2)	2.4	(0.6)	2.7	(0.7)	4.2	(1.7)
Balance_ mean (SD)	−1.91	(1.9)	−0.3	(0.7)	−1.6	(0.9)	−3.3	(1.5)	−5.6	(0.7)	−8.9	(0.9)	−9.8	(1.7)
Coverage %_ mean (SD)	65.50	(30.6)	90.5	(27)	62.5	(20.1)	46.5	(23.2)	30.3	(7.6)	23.6	(5.9)	29.8	(12.1)
Patient outcomes														
Mortality	3419	(1.9)	150	(0.2)	811	(1.6)	1695	(3.3)	734	(5.5)	21	(5.8)	8	(9.9)
Readmission (< 31 days)	8961	(4.9)	654	(1.0)	2497	(4.6)	3568	(7.0)	1317	(9.8)	20	(5.5)	5	(1.2)
Nurse-sensitive adverse event	29,158	(15.9)	3853	(5.6)	8062	(14.9)	13,656	(26.9)	3553	(26.6)	229	(63.1)	75	(92.6)
HCA infection^a^	9978	(5.5)	1083	(1.7)	2509	(4.6)	5257	(10.3)	1009	(7.5)	84	(23.1)	36	(44.4)
Surgical site infection	2092	(1.1)	513	(1.1)	899	(1.7)	642	(1.3)	26	(0.2)	10	(2.8)	2	(2.5)
CLABSI	537	(0.3)	20	(< 0.1)	113	(0.2)	344	(0.7)	46	(0.3)	12	(3.3)	2	(2.5)
MRO infection	6000	(3.3)	491	(0.8)	1312	(2.4)	3372	(6.6)	746	(5.6)	56	(15.4)	23	(28.4)
Urinary tract infection	747	(0.4)	69	(0.1)	67	(0.1)	556	(1.1)	44	(0.3)	7	(1.9)	4	(4.9)
Failure to maintain^b^	19,148	(10.5)	2508	(3.9)	5564	(10.3)	8151	(16.0)	2766	(20.9)	71	(19.6)	65	(80.2)
Aspiration pneumonia	1206	(0.7)	44	(< 0.1)	297	(0.6)	664	(1.3)	182	(1.4)	8	(2.2)	11	(13.6)
Delirium	3138	(1.7)	428	(0.7)	906	(1.8)	149	(2.9)	291	(2.2)	9	(2.5)	14	(17.3)
Pressure ulcer	1669	(0.9)	160	(0.2)	378	(0.7)	814	(1.6)	284	(2.1)	12	(3.3)	21	(25.9)
Incontinence	2282	(1.3)	136	(0.2)	447	(0.9)	600	(1.2)	105	(7.8)	5	(1.4)	44	(54.3)
Falls	1216	(0.7)	173	(0.3)	336	(0.6)	557	(1.1)	144	(1.1)	2	(0.6)	4	(4.9)
Phlebitis	9822	(5.4)	1527	(2.4)	3321	(6.1)	3913	(7.7)	1018	(7.6)	32	(8.8)	11	(13.6)
Hypostatic pneumonia	1478	(0.8)	146	(0.2)	390	(0.7)	728	(1.4)	206	(1.5)	5	(1.4)	3	(3.7)
Avoidable critical complications	5825	(3.2)	433	(0.7)	1309	(2.4)	3452	(7.0)	440	(3.3)	173	(47.7)	18	(22.2)
Cardiac arrest	386	(0.2)	40	(0.1)	106	(0.2)	192	(0.4)	46	(0.3)	1	(0.3)	1	(1.2)
Shock	1769	(0.9)	154	(0.2)	479	(0.9)	837	(1.6)	142	(1.1)	145	(39.9)	12	(14.8)
Thrombotic event	1148	(0.6)	78	(0.1)	259	(0.5)	725	(1.4)	77	(0.6)	8	(2.2)	1	(1.2)
Sepsis	3311	(1.8)	240	(0.4)	644	(1.2)^a^	2111	(4.2)	226	(1.7)	77	(21.2)	13	(16)

Abbreviations: *a*NHPPD = available RN hours per patient day, CLABSI = central line-associated bloodstream infection, HCA = healthcare-associated, ICU = intensive care unit, *r*NHPPD = required RN hours per patient day, SD = standard deviation.

^a^Included those patients with *Aspiration pneumonia* event.

^b^Included those patients with urinary tract infection event.

**Table 3:** Nurse staffing coverage and patient outcomes according to the reason for admission.

Nurse staffing coverage and outcomes	Nurse staffing coverage								Patient outcomes					
	*r*NHPPD		*a*NHPPD		Balance		Coverage		Readmission		Mortality		NSAE	
	Mean	SD	Mean	SD	Mean	SD	Mean	SD	*N*	(%)	*N*	(%)	*N*	(%)
Reason for admission														
Cardiocirculatory	4.9	(1.6)	2.8	(1.3)	−2.0	(2.2)	66.7	(44.8)	1374	(4.5)	540	(1.8)	4036	(13.3)
Infectious	6.0	(1.3)	2.4	(0.5)	−3.6	(1.4)	43.0	(15.0)	2056	(7.6)	718	(2.6)	8745	(32.1)
General surgery	3.5	(1.3)	2.5	(0.5)	−1.1	(1.3)	76.3	(24.1)	892	(4.3)	211	(1.0)	2188	(10.5)
Trauma and orthopedics	3.1	(0.8)	2.2	(0.4)	−0.8	(0.8)	75.2	(16)	374	(1.9)	144	(0.7)	1546	(7.8)
Digestive, liver, and pancreatic	4.7	(1)	2.6	(0.7)	−2.1	(1.2)	58.1	(18.3)	1387	(7)	462	(2.3)	3871	(19.6)
Nervous system	4.8	(1)	3.2	(1.8)	−1.5	(1.7)	69.5	(32.4)	557	(3.6)	507	(3.3)	2960	(19.1)
Kidney and urinary tract	3.7	(1.6)	2.5	(0.6)	−1.2	(1.6)	76.1	(23.9)	878	(6.3)	130	(0.9)	1489	(10.7)
Respiratory	6.3	(2)	2.4	(0.6)	−3.8	(2.2)	45.0	(21.5)	802	(7.3)	312	(2.8)	1807	(16.5)
Reproductive	2.8	(0.3)	2.6	(0.5)	−0.2	(0.5)	92.5	(17.1)	174	(2.1)	3	(0.0)	262	(3.2)
Head, neck, and maxillofacial	3	(0.6)	2.3	(0.4)	−0.7	(0.7)	77.9	(17.6)	132	(2.4)	11	(0.2)	309	(5.6)
Metabolic, nutritional, and endocrinology	3.6	(1.5)	2.5	(0.4)	−1.1	(1.7)	80.6	(29.9)	111	(3.6)	28	(0.9)	245	(8)
Hematopoiesis, blood, and immunologic	5.8	(0.6)	2.5	(0.5)	−3.3	(0.7)	43.8	(9.6)	241	(8.9)	85	(3.1)	552	(20.4)
Psychiatric, mental health, and addictions	3.2	(0.5)	3.4	(1.3)	0.2	(1.4)	107.9	(43.1)	30	(2.5)	2	(0.2)	151	(12.7)
Skin and burns	3.5	(0.9)	3.2	(0.9)	−0.4	(1.3)	95.1	(36.8)	22	(2.4)	7	(0.8)	109	(12)
Eyes	2.5	(0.1)	2.4	(0.3)	−0.1	(0.3)	95.8	(12.6)	7	(0.8)	2	(0.2)	34	(4)
Other conditions	7.6	(1.8)	2.4	(0.4)	−5.2	(1.8)	35.6	(18)	177	(8.2)	257	(12)	854	(39.7)

*Note:* Other conditions group mainly included admissions for unspecific clinical decline-related advanced neoplastic disease in fragile adults.

Abbreviations: *a*NHPPD = available RN hours per patient day, NSAE = nurse-sensitive adverse event, *r*NHPPD = required RN hours per patient day, SD = standard deviation.

**Table 4:** Safety levels of nurse staffing coverage and patient outcomes.

Outcomes	All		≤ 90 coverage		> 90 coverage		*p*-value
	*n* = 183,085		*n* = 145,093 (79.2)		*n* = 37,992 (20.8)		
	*N*	(%)	*N*	(%)	*N*	(%)	
Patient outcomes							
Mortality	3419	(1.9)	3055	(2)	364	(0.9)	< 0.001
Hospital readmission (< 31 days)	9214	(5)	8048	(5.5)	1166	(3.07)	< 0.001
Nurse-sensitive adverse event	29,158	(15.9)	25,282	(17.4)	3876	(10.2)	< 0.001
HCA infection^a^	9978	(5.5)	8806	(6.1)	1172	(3.1)	< 0.001
Surgical site infection	2092	(1.1)	1642	(1.1)	450	(1.2)	0.40
CLABSI	537	(0.3)	491	(0.3)	46	(0.1)	< 0.001
Multidrug-resistant organism infection	6000	(3.3)	5477	(3.8)	523	(1.4)	< 0.001
Urinary tract infection	747	(0.4)	693	(0.5)	54	(0.1)	< 0.001
Failure to maintain^b^	19,148	(10.5)	16,353	(11.3)	2795	(7.4)	< 0.001
Aspiration pneumonia	1206	(0.7)	1025	(0.7)	181	(0.5)	< 0.001
Delirium	3138	(1.7)	2555	(1.8)	583	(1.5)	0.003
Pressure ulcer	1669	(0.9)	1407	(1)	262	(0.7)	< 0.001
Incontinence	2282	(1.2)	2014	(1.4)	268	(0.7)	< 0.001
Falls	1216	(0.7)	1040	(0.7)	176	(0.5)	< 0.001
Venous catheter-related phlebitis	9822	(5.4)	8341	(5.8)	1481	(3.9)	< 0.001
Hypostatic pneumonia	1478	(0.8)	1318	(0.9)	160	(0.4)	< 0.001
Avoidable critical complications	5825	(3.2)	5204	(3.6)	621	(1.6)	< 0.001
Cardiac arrest	386	(0.2)	294	(0.2)	92	(0.2)	0.15
Shock	1769	(1)	1529	(1.1)	240	(0.6)	< 0.001
Thrombotic event	1148	(0.6)	1051	(0.7)	97	(0.3)	< 0.001
Sepsis	3311	(1.8)	3019	(2.1)	292	(0.8)	< 0.001

Abbreviations: CLABSI = central line-associated bloodstream infection, HA = hospital-acquired.

^a^Included those patients with *Aspiration pneumonia* event.

^b^Included those patients with HA urinary tract infection event.

**Table 5:** Association between nurse staffing coverage and patient outcomes.

Outcomes	Before adjustment		After adjustment	
	Risk ratio	95% CI	Risk ratio	95% CI
Mortality	0.44	0.42–0.46	0.41	0.37–0.45
Readmission	0.81	0.79–0.83	0.93	0.89–0.97
NSAE	0.57	0.56–0.58	0.67	0.66–0.69
Healthcare-associated infections	0.50	0.48–0.51	0.60	0.57–0.63
Failure to maintain	0.62	0.61–0.64	0.69	0.67–0.72
Avoidable critical complications	0.44	0.42–0.46	0.46	0.43–0.49

*Note:* Multivariate analysis was adjusted by age, gender, admission type, reason for admission, clinical assignment, and hospital characteristics.

Abbreviations: CI = confidence interval, NSAE = nurse-sensitive adverse event.

## Data Availability

All relevant data are within the manuscript. Complete dataset cannot be shared by the investigator because all data were collected from the electronic health record system with the authorization of the Catalan Institute of Health.

## Supporting Information

Appendix 1: ATIC patient classification system: Acuity clusters, subgroups, and equivalence to required nursing hours per patient day. Appendix 2: Detailed ICD-9 and ATIC terminology codes for nurse-sensitive adverse events. Appendix 3: Mortality and nurse-sensitive adverse event incidence curves according to the levels of nursing staffing coverage. Appendix 4: Additional considerations on selected nurse-sensitive adverse events.

## References

[1] Aiken L., Clarke S., Sloane D., Sochalski J., Silber J. (2002). Hospital Nurse Staffing and Patient Mortality, Nurse Burnout, and Job Dissatisfaction. *Journal of the American Medical Association*.

[2] Needleman J., Buerhaus P., Mattke S., Stewart M., Zelevinsky K. (2002). Nurse-Staffing Levels and the Quality of Care in Hospitals. *New England Journal of Medicine*.

[3] Needleman J., Buerhaus P., Pankratz V. S., Leibson C. L., Stevens S. R., Harris M. (2011). Nurse Staffing and Inpatient Hospital Mortality. *New England Journal of Medicine*.

[4] Aiken L. H., Sloane D. M., Bruyneel L. (2014). Nurse Staffing and Education and Hospital Mortality in Nine European Countries: A Retrospective Observational Study. *The Lancet*.

[5] Dall’Ora C., Saville C., Rubbo B., Turner L., Jones J., Griffiths P. (2022). Nurse Staffing Levels and Patient Outcomes: A Systematic Review of Longitudinal Studies. *International Journal of Nursing Studies*.

[6] McHugh M. D., Aiken L. H., Sloane D. M., Windsor C., Douglas C., Yates P. (2021). Effects of Nurse-To-Patient Ratio Legislation on Nurse Staffing and Patient Mortality, Readmissions, and Length of Stay: A Prospective Study in a Panel of Hospitals. *The Lancet*.

[7] Ball J. E., Bruyneel L., Aiken L. H. (2018). Post-Operative Mortality, Missed Care and Nurse Staffing in Nine Countries: A Cross-Sectional Study. *International Journal of Nursing Studies*.

[8] Griffiths P., Recio-Saucedo A., Dall’Ora C. (2018). The Association between Nurse Staffing and Omissions in Nursing Care: A Systematic Review. *Journal of Advanced Nursing*.

[9] Aiken L. H., Cimiotti J. P., Sloane D. M., Smith H. L., Flynn L., Neff D. F. (2011). Effects of Nurse Staffing and Nurse Education on Patient Deaths in Hospitals With Different Nurse Work Environments. *Medical Care*.

[10] Duffield C., Diers D., O’Brien-Pallas L. (2011). Nursing Staffing, Nursing Workload, the Work Environment and Patient Outcomes. *Applied Nursing Research*.

[11] Wei H., Sewell K. A., Woody G., Rose M. A. (2018). The State of the Science of Nurse Work Environments in the United States: A Systematic Review. *International Journal of Nursing Science*.

[12] Rodrigues L. P., de Oliveira Rezende A. T., Delpino F. M. (2022). Association Between Multimorbidity and Hospitalization in Older Adults: Systematic Review and Meta-Analysis. *Age and Ageing*.

[13] Legido-Quigley H., Berrojalbiz I., Franco M. (2024). Towards an Equitable People-Centred Health System for Spain. *The Lancet*.

[14] Saville C. E., Griffiths P., Ball J. E., Monks T. (2019). How Many Nurses Do We Need? A Review and Discussion of Operational Research Techniques Applied to Nurse Staffing. *International Journal of Nursing Studies*.

[15] Van den Heede K., Diya L., Lesaffre E., Vleugels A., Sermeus W. (2008). Benchmarking Nurse Staffing Levels: the Development of a Nationwide Feedback Tool. *Journal of Advanced Nursing*.

[16] Rafferty A. M., Clarke S. P., Coles J. (2007). Outcomes of Variation in Hospital Nurse Staffing in English Hospitals: Cross-Sectional Analysis of Survey Data and Discharge Records. *International Journal of Nursing Studies*.

[17] Oner B., Zengul F. D., Oner N., Ivankova N. V., Karadag A., Patrician P. A. (2021). Nursing‐Sensitive Indicators for Nursing Care: A Systematic Review (1997–2017). *Nursing Open*.

[18] Leary A., Punshon G. (2019). Determining Acute Nurse Staffing: A Hermeneutic Review of an Evolving Science. *BMJ Open*.

[19] Needleman J., Shekelle P. G. (2019). More Ward Nursing Staff Improves Inpatient Outcomes, But How Much is Enough?. *BMJ Quality and Safety*.

[20] Welton J. M. (2017). Measuring Patient Acuity: Implications for Nurse Staffing and Assignment. *The Journal of Nursing Administration: The Journal of Nursing Administration*.

[21] Kirby K. (2015). Hours Per Patient Day: Not the Problem, Nor the Solution. *Nursing Economics*.

[22] Kiekkas P., Tsekoura V., Aretha D. (2019). Nurse Understaffing is Associated With Adverse Events in Postanaesthesia Care Unit Patients. *Journal of Clinical Nursing*.

[23] Griffiths P., Maruotti A., Recio Saucedo A. (2019). Nurse Staffing, Nursing Assistants and Hospital Mortality: Retrospective Longitudinal Cohort Study. *BMJ Quality and Safety*.

[24] Shang J., Needleman J., Liu J., Larson E., Stone P. W. (2019). Nurse Staffing and Healthcare-Associated Infection, Unit-Level Analysis. *The Journal of Nursing Administration: The Journal of Nursing Administration*.

[25] Park C. S. (2017). Optimizing Staffing, Quality, and Cost in Home Healthcare Nursing: Theory Synthesis. *Journal of Advanced Nursing*.

[26] Juvé-Udina M. E., Adamuz J., López-Jimenez M. M. (2019). Predicting Patient Acuity According to Their Main Problem. *Journal of Nursing Management*.

[27] Armijo-Olivo S., Craig R., Corabian P., Guo B., Souri S., Tjosvold L. (2020). Nursing Staff Time and Care Quality in Long-Term Care Facilities: A Systematic Review. *The Gerontologist*.

[28] Jiménez-Martínez E., Adamuz J., González-Samartino M. (2024). Peripheral Intravenous Catheter Failure, Nurse Staffing Levels and Care Complexity Individual Factors: A Retrospective Multicentre Cohort Study. *PLoS One*.

[29] Twigg D., Duffield C. (2009). A Review of Workload Measures: A Context for a New Staffing Methodology in Western Australia. *International Journal of Nursing Studies*.

[30] Montalvo I. (2007). American Nurses Association Nursing Sensitive Measures National Database of Nursing Quality Indicators (NDNQI®). https://www.ncvhs.hhs.gov/wp-content/uploads/2014/05/070619p8.pdf.

[31] Horan T. C., Andrus M., Dudeck M. A. (2008). CDC/NHSN Surveillance Definition of Health Care–Associated Infection and Criteria for Specific Types of Infections in the Acute Care Setting. *American Journal of Infection Control*.

[32] Bail K., Grealish L. (2016). Failure to Maintain: A Theoretical Proposition for a New Quality Indicator of Nurse Care Rationing for Complex Older People in Hospital. *International Journal of Nursing Studies*.

[33] Mushta J., L Rush K., Andersen E. (2018). Failure to Rescue as a Nurse-Sensitive Indicator. *Nursing Forum*.

[34] World Health Organization (2018). Life Expectancy and Healthy Life Expectancy. http://apps.who.int/gho/data/view.main.SDG2016LEXREGv?lang=en.

[35] Eurostat (2019). Mortality and Life Expectancy Statistics. https://ec.europa.eu/eurostat/statistics-explained/index.php/Mortality_and_life_expectancy_statistics.

[36] Eurostat (2018). Health Statistics at Regional Level. https://ec.europa.eu/eurostat/statistics-explained/index.php/Health_statistics_at_regional_level.

[37] Coduras A., Llano Señarís J. (2018). La Sanidad Española en Cifras. https://www.aeesme.org/wp-content/uploads/2019/05/Sanidad-espanola-en-cifras-2018.pdf.

[38] Pearse R. M., Moreno R. P., Bauer P. (2012). Mortality After Surgery in Europe: A 7 Day Cohort Study. *The Lancet*.

[39] Bailey M., Weiss A., Barrett M., Jiang H. (2019). Statistical Brief # 248. Healthcare Cost and Utilization Project (HCUP). https://hcup-us.ahrq.gov/reports/statbriefs/sb248-Hospital-Readmissions-2010-2016.jsp.

[40] van Galen L. S., Brabrand M., Cooksley T. (2017). Patients’ and Providers’ Perceptions of the Preventability of Hospital Readmission: A Prospective, Observational Study in Four European Countries. *BMJ Quality and Safety*.

[41] Fischer C., Anema H. A., Klazinga N. S. (2012). The Validity of Indicators for Assessing Quality of Care: A Review of the European Literature on Hospital Readmission Rate. *The European Journal of Public Health*.

[42] Jiang H. J., Hensche M. (2016). Characteristics of 30-Day All-Cause Hospital Readmissions, 2016–2020. https://hcup-us.ahrq.gov/reports/statbriefs/sb304-readmissions-2016-2020.jsp.

[43] McHugh M. D., Berez J., Small D. S. (2013). Hospitals With Higher Nurse Staffing Had Lower Odds of Readmissions Penalties Than Hospitals With Lower Staffing. *Health Affairs*.

[44] Giuliano K. K., Danesh V., Funk M. (2016). The Relationship between Nurse Staffing and 30-Day Readmission for Adults With Heart Failure. *The Journal of Nursing Administration: The Journal of Nursing Administration*.

[45] Kim S. J., Park E.-C., Han K.-T., Kim S. J., Kim T. H. (2016). Nurse Staffing and 30-Day Readmission of Chronic Obstructive Pulmonary Disease Patients: A 10-Year Retrospective Study of Patient Hospitalization. *Asian Nursing Research*.

[46] Adamuz J., González-Samartino M., Jiménez-Martínez E. (2018). Care Complexity Individual Factors Associated With Hospital Readmission: A Retrospective Cohort Study. *Journal of Nursing Scholarship*.

[47] Twigg D. E., Gelder L., Myers H. (2015). The Impact of Understaffed Shifts on Nurse‐Sensitive Outcomes. *Journal of Advanced Nursing*.

[48] Kim C.-G., Bae K.-S. (2018). Relationship Between Nurse Staffing Level and Adult Nursing-Sensitive Outcomes in Tertiary Hospitals of Korea: Retrospective Observational Study. *International Journal of Nursing Studies*.

[49] McHugh M. D., Rochman M. F., Sloane D. M. (2016). Better Nurse Staffing and Nurse Work Environments Associated With Increased Survival of In-Hospital Cardiac Arrest Patients. *Medical Care*.

[50] Cho E., Chin D. L., Kim S., Hong O. (2016). The Relationships of Nurse Staffing Level and Work Environment With Patient Adverse Events. *Journal of Nursing Scholarship*.

[51] Shin S., Park J., Bae S. (2019). Nurse Staffing and Hospital‐Acquired Conditions: A Systematic Review. *Journal of Clinical Nursing*.

[52] Haque M., Sartelli M., McKimm J., Abu Bakar M. B. (2018). Health Care-Associated Infections–An Overview. *Infection and Drug Resistance*.

[53] Khan H. A., Baig F. K., Mehboob R. (2017). Nosocomial Infections: Epidemiology, Prevention, Control and Surveillance. *Asian Pacific Journal of Tropical Biomedicine*.

[54] Cassini A., Plachouras D., Eckmanns T. (2016). Burden of Six Healthcare-Associated Infections on European Population Health: Estimating Incidence-Based Disability-Adjusted Life Years Through a Population Prevalence-Based Modelling Study. *PLoS Medicine*.

[55] van Mourik M. S. M., van Duijn P. J., Moons K. G. M., Bonten M. J. M., Lee G. M. (2015). Accuracy of Administrative Data for Surveillance of Healthcare-Associated Infections: A Systematic Review. *BMJ Open*.

[56] Mitchell B. G., Gardner A., Stone P. W., Hall L., Pogorzelska-Maziarz M. (2018). Hospital Staffing and Health Care–Associated Infections: A Systematic Review of the Literature. *Joint Commission Journal on Quality and Patient Safety*.

[57] Cho S.-H., Yun S.-C. (2009). Bed-to-nurse Ratios, Provision of Basic Nursing Care, and In-Hospital and 30-Day Mortality Among Acute Stroke Patients Admitted to an Intensive Care Unit: Cross-Sectional Analysis of Survey and Administrative Data. *International Journal of Nursing Studies*.

[58] Stalpers D., de Brouwer B. J. M., Kaljouw M. J., Schuurmans M. J. (2015). Associations between Characteristics of the Nurse Work Environment and Five Nurse-Sensitive Patient Outcomes in Hospitals: A Systematic Review of Literature. *International Journal of Nursing Studies*.

[59] He J., Staggs V. S., Bergquist-Beringer S., Dunton N. (2016). Nurse Staffing and Patient Outcomes: A Longitudinal Study on Trend and Seasonality. *BMC Nursing*.

[60] Nogueira T. d A., Menegueti M. G., Perdoná G. D. S. C., Auxiliadora-Martins M., Fugulin F. M. T., Laus A. M. (2017). Effect of Nursing Care Hours on the Outcomes of Intensive Care Assistance. *PLoS One*.

[61] Juvé-Udina M. E., Fabrellas-Padrés N., Adamuz-Tomás J. (2017). Surveillance Nursing Diagnoses, Ongoing Assessment and Outcomes on In-Patients Who Suffered a Cardiorespiratory Arrest. *Revista da Escola de Enfermagem da USP*.

[62] Kang C., Ryu H. G. (2023). Impact of Institutional Case Volume on Intensive Care Unit Mortality. *Acute and Critical Care*.

[63] Min A., Scott L. D. (2016). Evaluating Nursing Hours Per Patient Day as a Nurse Staffing Measure. *Journal of Nursing Management*.

[64] Aiken L. H., Sloane D., Griffiths P. (2017). Nursing Skill Mix in European Hospitals: Cross-Sectional Study of the Association With Mortality, Patient Ratings, and Quality of Care. *BMJ Quality and Safety*.

[65] Garcia A. L. (2017). Variability in Acuity in Acute Care. *The Journal of Nursing Administration: The Journal of Nursing Administration*.

[66] Bae S. (2022). Noneconomic and Economic Impacts of Nurse Turnover in Hospitals: A Systematic Review. *International Nursing Review*.

[67] Kunaviktikul W., Wichaikhum O., Nantsupawat A. (2015). Nurses’ Extended Work Hours: Patient, Nurse and Organizational Outcomes. *International Nursing Review*.

[68] Griffiths P., Saville C., Ball J. E., Jones J., Monks T. (2021). Beyond Ratios-Flexible and Resilient Nurse Staffing Options to Deliver Cost-Effective Hospital Care and Address Staff Shortages: A Simulation and Economic Modelling Study. *International Journal of Nursing Studies*.

[69] van der Mark C. J. E. M., Vermeulen H., Hendriks P. H. J., Oostveen C. J. van (2021). Measuring Perceived Adequacy of Staffing to Incorporate Nurses’ Judgement into Hospital Capacity Management: A Scoping Review. *BMJ Open*.

[70] Juvé-Udina M.-E., Adamuz J. (2023). Nursing Knowledge Tools and Strategies to Improve Patient Outcomes and the Work Environment. *Mentoring in Nursing through Narrative Stories Across the World*.

[71] Kwon H., Lee D. (2024). Clinical Decision Support System for Clinical Nurses’ Decision-Making on Nurse-To-Patient Assignment: a Scoping Review Protocol. *BMJ Open*.

[72] Kim J., Kang T., Seo H. J. (2022). Measuring Patient Acuity and Nursing Care Needs in South Korea: Application of a New Patient Classification System. *BMC Nursing*.

[73] González-Samartino M., Delgado-Hito P., Adamuz-Tomás J., Cano M. F. V., Creus M. C., Juvé-Udina M. E. (2018). Accuracy and Completeness of Records of Adverse Events Through Interface Terminology. *Revista da Escola de Enfermagem da USP*.

[74] Blauw M., Foxman B., Wu J., Rey J., Kothari N., Malani A. N. (2019). Risk Factors and Outcomes Associated With Hospital-Onset Peripheral Intravenous Catheter–Associated *Staphylococcus aureus* Bacteremia. *Open Forum Infectious Diseases*.

[75] Chopra V., Anand S., Hickner A. (2013). Risk of Venous Thromboembolism Associated With Peripherally Inserted Central Catheters: A Systematic Review and Meta-Analysis. *The Lancet*.

[76] Mino J. S., Gutnick J. R., Monteiro R., Anzlovar N., Siperstein A. E. (2014). Line-Associated Thrombosis as the Major Cause of Hospital-Acquired Deep Vein Thromboses: An Analysis From National Surgical Quality Improvement Program Data and a Call to Reassess Prophylaxis Strategies. *The American Journal of Surgery*.

[77] Park C. S. (2023). More is Not Always Better: Park’s Sweet Spot Theory‐Driven Implementation Strategy for Viable Optimal Safe Nurse Staffing Policy in Practice. *International Nursing Review*.

[78] Griffiths P., Saville C., Ball J. (2023). Costs and Cost-Effectiveness of Improved Nurse Staffing Levels and Skill Mix in Acute Hospitals: A Systematic Review. *International Journal of Nursing Studies*.

[79] Castellà-Creus M., Delgado-Hito P., Casanovas-Cuellar C., Tàpia-Pérez M., Juvé-Udina M. E. (2019). Barriers and Facilitators Involved in Standardised Care Plan Individualisation Process in Acute Hospitalisation Wards: A Grounded Theory Approach. *Journal of Clinical Nursing*.

