# Supplementary material for: Association Between Nurse Staffing Coverage and Patient Outcomes in a Context of Prepandemic Structural Understaffing: A Patient-Unit-Level Analysis
Source: J Nurs Manag. 2025 Feb 24;2025:8003569. doi: 10.1155/jonm/8003569 (PMC11985225; doi:10.1155/jonm/8003569)
Supplement: Supporting Information — Additional supporting information can be found online in the Supporting Information section. [file 8003569.f1.docx]

Appendix 1. ATIC Patient Classification system: Acuity clusters, subgroups and equivalence to required nursing hours per patient day.

|  | | | | | | |
| --- | --- | --- | --- | --- | --- | --- |
| **Acuity cluster** | **Weight range** | **NP ratio** | **NHPPD** | **NMPPD** | **NHPPS** | **NMPPS** |
| **Gigaintensive** | **900-1000** | **≥ 2 : 1** | **31 - 42h** | **1860-2520** | **11-14h** | **620-840** |
|  | 976-1000 |  | 42 | 2520 | 14 | 840 |
|  | 951-975 |  | 38 | 2280 | 12.6 | 760 |
|  | 926-950 |  | 34 | 2040 | 11.3 | 680 |
|  | 901-925 |  | 31 | 1860 | 10.3 | 620 |
| **Megaintensive** | **801-900** | **1.5 : 1** | **21 - 30h** | **1260-1800** | **7 - 10h** | **480-660** |
|  | 876-900 |  | 30 | 1800 | 10 | 600 |
|  | 851-875 |  | 27 | 1620 | 9 | 540 |
|  | 826-850 |  | 24 | 1440 | 8 | 480 |
|  | 801-825 |  | 21 | 1260 | 7 | 420 |
| **Superintensive** | **701-800** | **1 : 1** | **14 - 20h** | **840-1200** | **4.6 - 6.6h** | **280-400** |
|  | 776-800 |  | 20 | 1200 | 6.6 | 400 |
|  | 751-775 |  | 18 | 1080 | 6 | 360 |
|  | 726-750 |  | 16 | 960 | 5.3 | 320 |
|  | 701-725 |  | 14 | 840 | 4.7 | 280 |
| **Intensive** | **601-700** | **1 : 2** | **10 - 13h** | **600-780** | **3.3 - 4.5h** | **200-260** |
|  | 676-700 |  | 13 | 780 | 4.3 | 260 |
|  | 651-675 |  | 12 | 720 | 4 | 240 |
|  | 626-650 |  | 11 | 660 | 3.7 | 220 |
|  | 601-625 |  | 10 | 600 | 3.3 | 200 |
| **Preintensive** | **501-600** | **1 : 3** | **7 - 10h** | **450-540** | **2.5 – 3h** | **150-180** |
|  | 576-600 |  | 9.75 | 585 | 3.2 | 195 |
|  | 551-575 |  | 9 | 540 | 3 | 180 |
|  | 526-550 |  | 8.25 | 495 | 2.7 | 165 |
|  | 501-525 |  | 7.5 | 450 | 2.5 | 150 |
| **Intermediate** | **401-500** | **1 : 4** | **5 - 7h** | **330-420** | **1.8 - 2.3h** | **110-140** |
|  | 476-500 |  | 7 | 420 | 2.3 | 140 |
|  | 451-475 |  | 6.5 | 390 | 2.2 | 130 |
|  | 426-450 |  | 6 | 360 | 2 | 120 |
|  | 401-425 |  | 5.5 | 330 | 1.8 | 110 |
| **Intensification** | **301-400** | **1 : 6** | **3 - 5h** | **210-300** | **1.2 - 1.7h** | **70-100** |
|  | 376-400 |  | 5 | 300 | 1.7 | 100 |
|  | 351-375 |  | 4.5 | 270 | 1.5 | 90 |
|  | 326-350 |  | 4 | 240 | 1.3 | 80 |
|  | 301-325 |  | 3.5 | 210 | 1.2 | 70 |
| **Acute** | **201-300** | **1 : 8** | **2 - 3h** | **135-180** | **0.8 – 1h** | **45-60** |
|  | 276-300 |  | 3 | 180 | 1 | 60 |
|  | 251-275 |  | 2.75 | 165 | 0.9 | 55 |
|  | 226-250 |  | 2.5 | 150 | 0.8 | 50 |
|  | 201-225 |  | 2.25 | 135 | 0.8 | 45 |
| **Subacute** | **101-200** | **1 : 12** | **1 - 2h** | **75-120** | **0.4 - 0.7h** | **25-40** |
|  | 176-200 |  | 2 | 120 | 0.7 | 40 |
|  | 151-175 |  | 1.75 | 105 | 0.6 | 35 |
|  | 126-150 |  | 1.5 | 90 | 0.5 | 30 |
|  | 101-125 |  | 1.25 | 75 | 0.4 | 25 |
| **Occasional** | **1-100** | **1 : 20** | **0.1-1h** | **15-60** | **0.1 - 0.3h** | **5-20** |
|  | 76-100 |  | 1 | 60 | 0.3 | 20 |
|  | 51-75 |  | 0.75 | 45 | 0.3 | 15 |
|  | 26-50 |  | 0.5 | 30 | 0.2 | 10 |
|  | 1-25 |  | 0.25 | 15 | 0.1 | 5 |
| Abbreviations: NP ratio, nurse per patient ratio; NHPPD, nursing hours per patient day; NMPPD, nursing minutes per patient day; NHPPS, nursing hours per patient shift; NMPPS, nursing minutes per patient shift  h, hours.  ATIC, *Acute to Intensive Care* Patient Classification System. This PCS derives from the patient main problem weight model based on nurses’ clinical judgments of the patient status and progress, using the ATIC Terminology. For this reason, the PCS adopts the ATIC acronym. | | | | | | |

Appendix 2. Detailed ICD-9 and ATIC terminology codes for nurse-sensitive adverse events

| ***HCA Infections*** | **ICD-9 Code** | **ICD-9 Label** | **ATIC v4.2 Code** | **ATIC v4.2 Label** |
| --- | --- | --- | --- | --- |
| Surgical site infection | **998.59** | **Other postoperative infection** | 10008904 | Surgical site infection |
| Central line-associated bloodstream infection | **999.31**  **996.62** | **Bloodstream infection due to central venous catheter**  **Infection and inflammatory reaction due to other vascular device, implant and graft.** | 10015574 | Venous catheter-associated bloodstream infection |
| MRO infection requiring isolation precautions | V07.0  V09.1 - .9  041.12  041.3  041.4  041.84  041.85 | Need for isolation and other prophylactic measures  Infection with microorganisms resistant to other specified drugs  MRSA in conditions classified elsewhere and of unspecified site  Klebsiella pneumoniae  Escherichia coli infections in conditions classified elsewhere and of unspecified site  Other specified bacterial infections in conditions classified elsewhere and of unspecified site, other anaerobes  Other specified bacterial infections in conditions classified elsewhere and of unspecified site, other gram-negatives | **10005773**  **10000104**  **10000107**  **10000106** | **Communicable disease +**  **Airborne precautions *or***  **Droplet precautions *and/or***  **Contact precautions** |
| Urinary tract infection | **996.64** | **Infection and inflammation due to indwelling urinary catheter** | 10006777  10015576 | Urinary tract infection  Urinary catheter-related infection |
| ***Failure to maintain*** | **ICD-9 Code** | **ICD-9 Label** | **ATIC v4.2 Code** | **ATIC v4.2 Label** |
| Aspiration pneumonia | **507.0 - .8** | **Pneumonitis due to inhalation food or vomitus** | 10015575 | Aspiration pneumonia |
| Hypostatic pneumonia | **514**  **518.0** | **Pulmonary congestion and hypostasis**  **Pulmonary collapse** | 10007143  10000406 | Hypostatic pneumonia  Atelectasis |
| Delirium | 293.0  292.81E  780.09  293.9  290.11  290.3  290.41 | Acute delirium  Drug-induced delirium  Delirium not otherwise specified  Postoperative acute confusional state  Presenile dementia with delirium  Senile dementia with delirium  Arteriosclerotic dementia with acute confusional state | **10001282**  **10005793**  **10000071**  **10001322** | **Delirium**  **Acute confusion**  **Psychomotor agitation**  **Disorientation** |
| Pressure ulcers | 707.00 - .15  707.20  707.21  707.22  707.23  707.24  707.25 | Pressure ulcer  Pressure ulcer unspecified stage  Pressure ulcer stage I  Pressure ulcer stage II  Pressure ulcer stage III  Pressure ulcer stage IV  Pressure ulcer unstageable | **10004898**  **10006092**  **10006093**  **10004899**  **10006094**  **10006095**  **10004900**  **10006096**  **10006097**  **10004901**  **10006098**  **10006099**  **10008129** | **Pressure ulcer stage 1**  **Pressure injury stage 1**  **Decubitus ulcer stage 1**  **Pressure ulcer stage 2**  **Pressure injury stage 2**  **Decubitus ulcer stage 2**  **Pressure ulcer stage 3**  **Pressure injury stage 3**  **Decubitus ulcer stage 3**  **Pressure ulcer stage 4**  **Pressure injury stage 4**  **Decubitus ulcer stage 4**  **Unstageable pressure ulcer** |
| Falls | E880  E884  E885  E886  E887  E888 | Fall on or from stairs or steps  Other fall from one level to another  Falls on same level from slipping, tripping or stumbling  Falls on the same level from collision, pushing or shoving by or with other person  Fracture, unspecified cause  Other or unspecified falls | **10000582**  **10007835**  **10006754**  **10007834**  **10006755**  **10006756**  **10007836**  **10008454** | **Fall**  **Fall without visible injury**  **Fall with minor injury**  **Fall with multiple minor injuries**  **Fall with moderate injury**  **Fall with severe injury**  **Fall with temporary loss of awareness**  **Fall with multiple injuries** |
| Incontinence | 788.91  788.31 - .33  788.32 – 625.6  788.33  787.60 | Functional urinary incontinence  Urge urinary incontinence (male / female)  Stress urinary incontinence (male / female)  Mixed urinary incontinence (urge and stress)  Fecal incontinence | **10002707**  **10007385**  **10007798**  **10007796**  **10007797**  **10007389**  **10002703**  **10007807**  **10007804**  **10007802**  **10007803**  **10007806**  **10002709**  **10005931**  **10007801**  **10007799**  **10007800** | **Urinary incontinence**  **Bladder incontinence**  **Passive (functional) urinary incontinence**  **Urge urinary incontinence**  **Stress urinary incontinence**  **Urinary leaks**  **Fecal incontinence**  **Bowel incontinence**  **Passive fecal incontinence**  **Urge fecal incontinence**  **Stress fecal incontinence**  **Fecal leaks**  **Urinofecal incontinence**  **Mixed incontinence**  **Passive urinofecal incontinence**  **Urge urinofecal incontinence**  **Stress urinofecal incontinence** |
| Venous catheter-related phlebitis | 451.9 | Phlebitis and thrombophlebitis unspecified | **10005869**  **10005870**  **10001284 +/-**  **10010467**  **10010468**  **10010469**  **10010470** | **Infusion phlebitis**  **Drug perfusion phlebitis**  **Venous catheter-related phlebitis**  **Unknown origin phlebitis**  **Probable infectious origin**  **Probable chemical origin**  **Probable mechanical origin** |
| ***Avoidable critical complications*** | **ICD-9 Code** | **ICD9 Label** | **ATIC v4.2 Code** | **ATIC v4.2 Label** |
| Shock | **249.8**  **249.3**  **250.3**  **995.0**  **977.9**  **995.60 - .60**  **999.41**  **785.50**  **998.0**  **785.51**  **785.52**  **995.92**  **785.59** | **Hypoglycemic coma**  **Secondary diabetes mellitus with other coma**  **Diabetes with other coma**  **Other anaphylactic reaction**  **Overdose or wrong substance given or taken NEC**  **Anaphylactic reaction due to unspecified food / specified food**  **Anaphylactic reaction due to administration of blood and blood products**  **Shock NOS**  **Postoperative shock NES**  **Cardiogenic shock**  **Septic shock**  **Severe sepsis**  **Other shock without mention of trauma** | 10015577  10015140  10000787  10006759  10006760  10006126  10006768  10006765  10006129 | Hypoglycemic shock  Hypoglycemic coma  Ketoacidosis  Anaphylactic shock  Cardiogenic shock  Cardiocirculatory collapse  Septic shock  Hypovolemic shock  Hemorrhagic shock |
| Cardiac arrest | **427.5** | **Cardiac arrest** | 10005710  10000411 | Cardiac arrest  Cardiorespiratory arrest |
| Thrombotic event | **451.1 - .2**  **451.81 - .89**  **453.40 - .42**  **453.8 - .89**  **453.9**  **415.11**  **415.13**  **415.19** | **Phlebitis and thrombophlebitis of deep veins of lower extremities / unspecified**  **Phlebitis and thrombophlebitis of iliac veins / deep veins of upper extremities / other sites**  **Acute venous embolism and thrombosis of unspecified deep vessels of lower extremity / proximal lower extremity / distal lower extremity**  **Acute venous embolism and thrombosis other specified veins /of deep veins of upper extremity / unspecified / axillary veins /subclavian veins / internal jugular veins / other thoracic veins**  **Other venous embolism and thrombosis of unspecified site**  **Iatrogenic pulmonary embolism and infarction**  **Saddle embolus of pulmonary artery**  **Other pulmonary embolism and infarction** | 10005868  10002345  10005564  10005563  10015579 | Thrombophlebitis  Phlebitis  Thrombosis  Thromboembolism  Pulmonary embolism |
| Sepsis | **038.0**  **038.1**  **038.2**  **038.3**  **038.4**  **038.8**  **038.9**  **790.7** | **Streptococcal septicemia**  **Staphylococcal septicemia**  **Pneumococcal septicemia**  **Septicemia due to anaerobes**  **Septicemia due to other gram-negative organisms**  **Other specified septicemias**  **Unspecified septicemia**  **Bacteremia** |  |  |

^a^ Not present on admission day

Codes used to collect patient data in the study appear in bold. ICD-9 data were obtained from the hospitals minimum data set, as secondary diagnosis. ATIC terminology data were obtained from the patients’ electronic health record system (original source) through the clinical data warehouse (study source). All were checked for *not present on admission* criterion.

Appendix 3. Outcomes incidence curves

Figure 1. Mortality at different levels of nurse staffing coverage

% Coverage


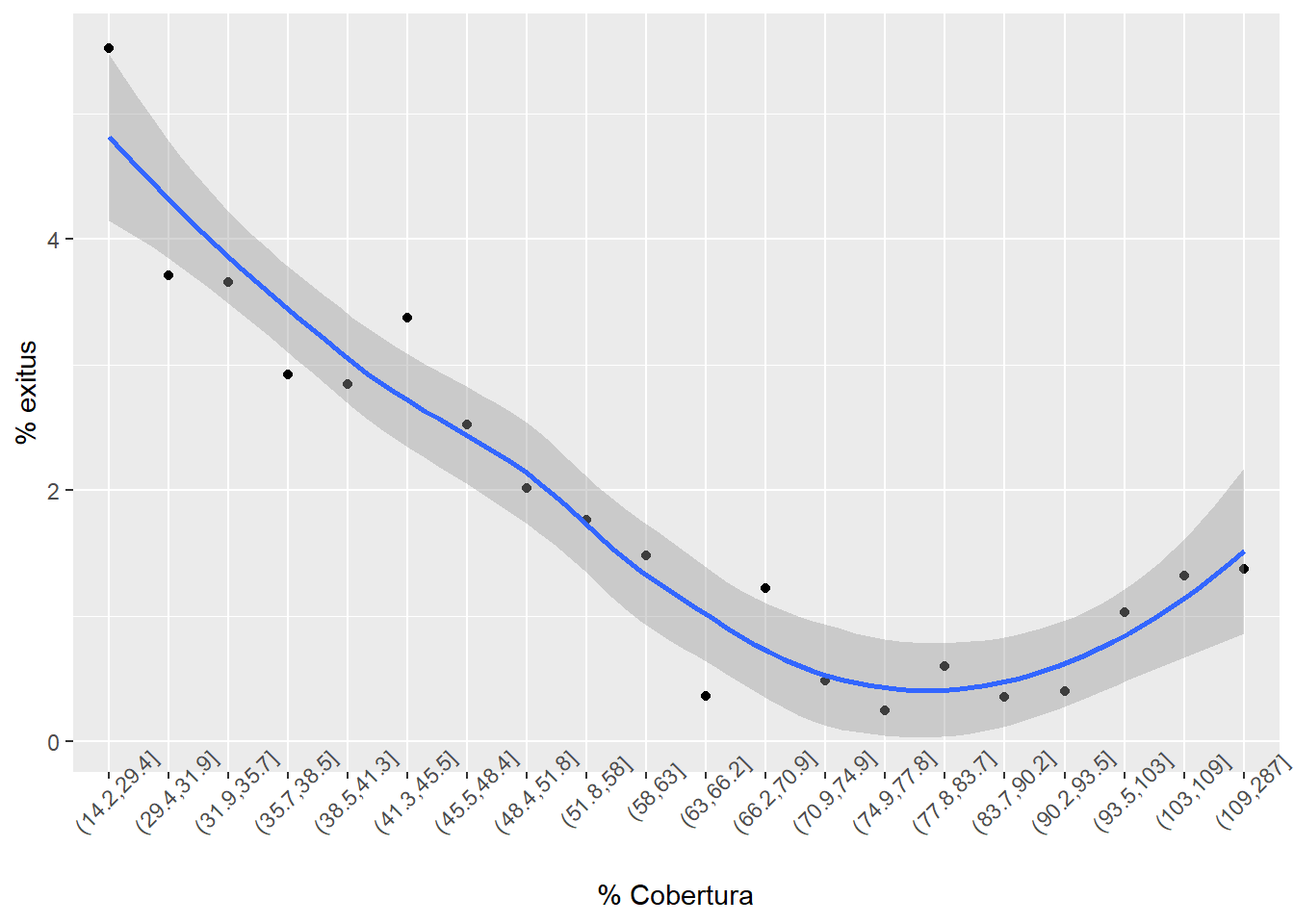


% Exitus

Figure 2. Nurse-sensitive adverse events at different levels of nurse staffing coverage


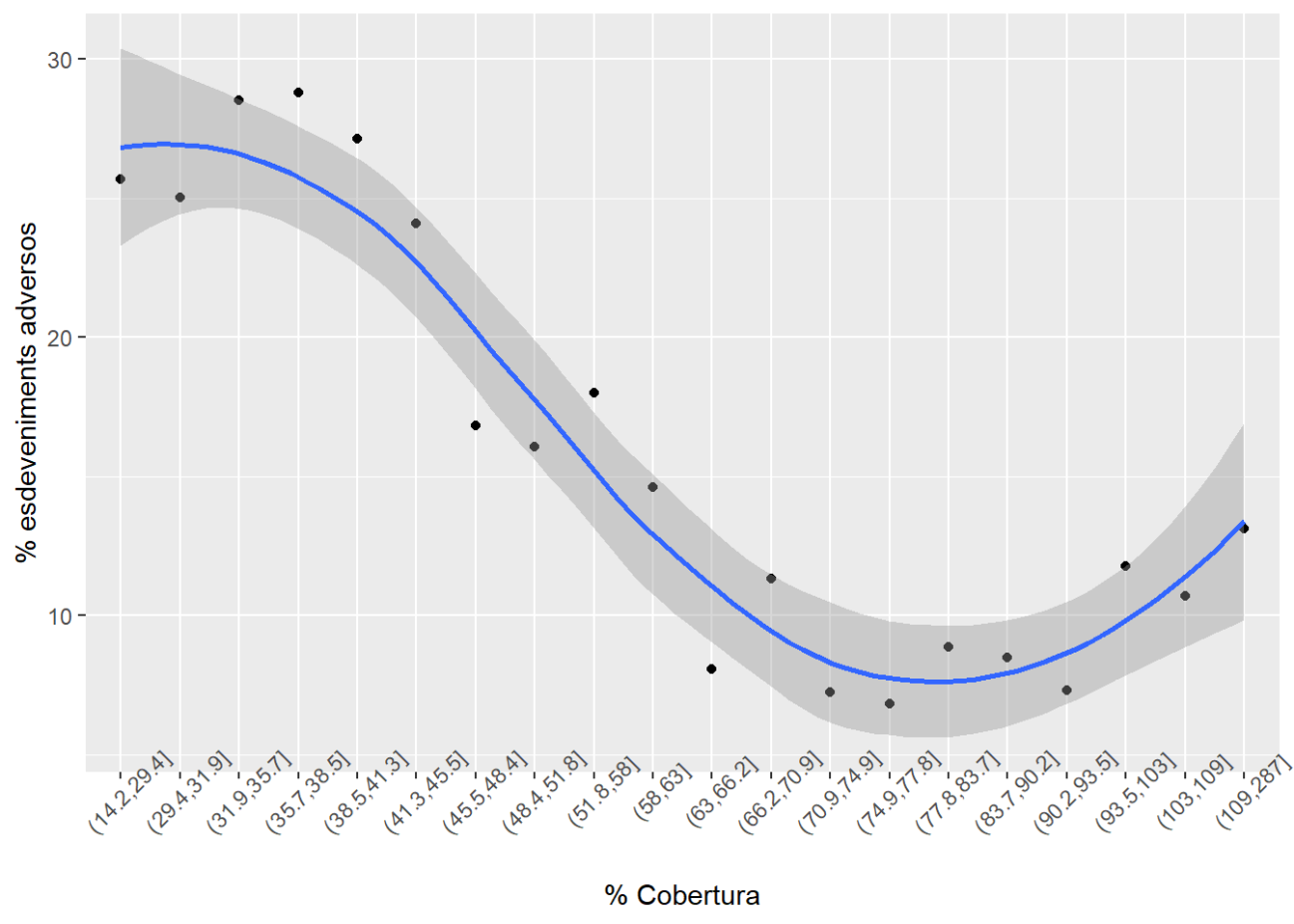


% Coverage

% Adverse events

Appendix 4. Additional considerations on selected nurse-sensitive adverse events.

**Urinary tract infections**

This study detailed urinary tract infections (UTI) findings at healthcare acquired infection cluster, but they were accounted into the failure to maintain cluster too. UTI is the fourth most common healthcare acquired infection (12%) and although 80% of in-hospital UTI affect patients with indwelling urinary catheter, this study considered not present on admission catheter-associated urinary tract infection (CAUTI) as well as UTI due to unknown cause or suspected cross-contamination with sponges or gloves during hygiene procedures, contaminated condoms, or related to other urinary devices like nephrostomy tubes or suprapubic catheters. Despite this, the frequency of UTI in this study is extremely low (0.4% in the overall population and 1.95% accounting only for patients with indwelling urinary catheter), indicating missed coding of this event in the hospital minimum data sets.

Nurse understaffing has been associated to CAUTI.

**Pneumonia**

Previous inquires shows that approximately 1.6% of hospitalized patients experience non-ventilator healthcare-associated pneumonia, with mortality rates ranging from 15-30%. Therefore, pneumonia is one of the most common health-associated infections. Recently, CDC published the oral health in healthcare setting to prevent pneumonia toolkit that suggest interventions can reduce a patient’s risk of developing non-ventilation healthcare-associated pneumonia. The results of previous studies show that improving oral care in hospitalized patients potentially reduced pneumonia rates. Therefore, although the original proposal for failure to maintain neither included a clear definition on pneumonia, there are numerous studies that shows the effectiveness of preventive interventions; so the present inquiry considered hypostatic pneumonia and atelectasis known to be preventable with patient repositioning, early mobilizing, pain control and breathing exercises, as well as aspiration pneumonia, potentially preventable with proper feeding care, comprehensive mouth care and oropharyngeal secretion management, swallowing screening and training, and respiratory surveillance and care. Aspiration pneumonia has been identified as a host factor associated to mortality in patients with healthcare acquired infections.

**Delirium**

Delirium has been related to increased mortality and longer hospital stay however, although nursing assessment and non-pharmacological interventions are effective in preventing incident delirium, no evidence on the effect of nurse staffing in delirium have been located.

**Pressure ulcers**

Contradicting results were observed for measures of nurse staffing and pressure injuries, with studies demonstrating better staffing associated to fewer pressure injuries but with small effect size, in contrast to large effect size in cohort studies revealing high staffing associated to high frequency of pressure sores. Later multicenter studies found weak or absent association between better staffing and reduction of pressure injuries, while a longitudinal inquiry found significant association considering both, trend, and seasonality.

**Persistent incontinence**

No studies on the association between nursing staffing and healthcare-acquired incontinence have been identified. It is estimated that 20%-24% of inpatients become incontinent during hospitalization however, our findings are significantly lower, even considering only patients aged over 75. Although adult diaper use may be recommended when patients are usual incontinent or severely injured, an overlooked, extended, random use of diapers in most bedridden patients might be existing, hindering the maintenance of out-of-diaper continence skills and having a decremental effect on the use of the toilet. In a recent study, unjustified use of adult diapers was 38%, but this value might be underrating the issue. Bathroom assistance was identified as the more frequent reason for patient-initiated call lights and one of the most common nursing care inpatient complaints. Indiscriminate use of disposable diapers is a signal of missed nursing care, since it is said to *save* time invested in bedpan assistance procedures, toileting activities and transfers or bedlinen changes, as well as in implementing routine screening for persistent incontinence. Furthermore, it could be contributing to trigger other complications like skin injuries, including pressure ulcers and diaper dermatitis, low self-esteem and hopelessness, self-imposed fluid intake limitation and dehydration, or UTI from contact with E. coli fecal flora in the diaper.

**Falls**

In this study, frequency findings on injurious and non-injurious inpatient falls (3.1 per 1,000 patient days) are consistent with published fall rates 3.5 falls per 1,000 inpatient days.

Regardless the staffing measure used, either nurse-patient ratio or NHPPD, and the country income level of the study setting, higher nursing staffing has been associated to fewer inpatient falls.

**Catheter-related phlebitis**

Frequency findings on this outcome in this study are close to the acceptable value for infusion phlebitis (5.5% vs 5%). Point prevalence of peripheral line phlebitis was estimated 3% to 12% in an international study and 8.4 per 1000 patient days.

It might be discussed whether infusion phlebitis should be considered within the failure to maintain cluster, or it might better match a new cluster grouping preventable non-infectious complications from therapeutic devices, sub-grouped as vascular access devices complications. This further reinforces the need to advance in a better refinement of nurse-sensitive outcomes. Two prior studies in hospital wards and intensive care setting demonstrate the association between catheter related phlebitis and mean nursing care hours.
